# Gluco-Incretins Regulate Beta-Cell Glucose Competence by Epigenetic Silencing of *Fxyd3* Expression

**DOI:** 10.1371/journal.pone.0103277

**Published:** 2014-07-24

**Authors:** David Vallois, Guy Niederhäuser, Mark Ibberson, Vini Nagaray, Lorella Marselli, Piero Marchetti, Jean-Yves Chatton, Bernard Thorens

**Affiliations:** 1 Center for Integrative Genomics, University of Lausanne, Lausanne, Switzerland; 2 Vital-IT group, SIB Swiss Institute of Bioinformatics, Lausanne, Switzerland; 3 Lund University Diabetes Center, Malmö, Sweden; 4 Department of Endocrinology and Metabolism, Ospedale di Cisanello, Pisa, Italy; 5 Department of Cell Biology and Morphology, University of Lausanne, Lausanne, Switzerland; CRCHUM-Montreal Diabetes Research Center, Canada

## Abstract

**Background/Aims:**

Gluco-incretin hormones increase the glucose competence of pancreatic beta-cells by incompletely characterized mechanisms.

**Methods:**

We searched for genes that were differentially expressed in islets from control and *Glp1r^−/−^; Gipr^−/−^* (dKO) mice, which show reduced glucose competence. Overexpression and knockdown studies; insulin secretion analysis; analysis of gene expression in islets from control and diabetic mice and humans as well as gene methylation and transcriptional analysis were performed.

**Results:**

*Fxyd3* was the most up-regulated gene in glucose incompetent islets from dKO mice. When overexpressed in beta-cells *Fxyd3* reduced glucose-induced insulin secretion by acting downstream of plasma membrane depolarization and Ca^++^ influx. *Fxyd3* expression was not acutely regulated by cAMP raising agents in either control or dKO adult islets. Instead, expression of *Fxyd3* was controlled by methylation of CpGs present in its proximal promoter region. Increased promoter methylation reduced *Fxyd3* transcription as assessed by lower abundance of H3K4me3 at the transcriptional start site and in transcription reporter assays. This epigenetic imprinting was initiated perinatally and fully established in adult islets. Glucose incompetent islets from diabetic mice and humans showed increased expression of *Fxyd3* and reduced promoter methylation.

**Conclusions/Interpretation:**

Because gluco-incretin secretion depends on feeding the epigenetic regulation of *Fxyd3* expression may link nutrition in early life to establishment of adult beta-cell glucose competence; this epigenetic control is, however, lost in diabetes possibly as a result of gluco-incretin resistance and/or de-differentiation of beta-cells that are associated with the development of type 2 diabetes.

## Introduction

The gluco-incretin hormones GLP-1 and GIP play multiple roles in the control of glucose homeostasis, in part by acting on pancreatic beta-cells. They potentiate glucose-induced insulin secretion (GIIS) [1], [2], induce beta-cell proliferation [3], [4], protect these cells against cytokine- or glucolipotoxicity-induced apoptosis [5], [6], and increase their glucose competence [7]. Their actions depend on their binding to specific Gs protein-coupled receptors [8], [9], which induce the production of cAMP leading to activation of protein kinase A, or of the cAMP binding protein Epac2 [10]. Intracellular signaling of the GLP-1 receptor also includes interaction with β-arrestins [11]–[13]. An important component of the action of GLP-1 is the induction of IGF-1R and IRS-2 expression and activation of the PI3K/Akt signaling pathway by autocrine secretion of IGF-2 and its binding to the IGF-1R [7], [14], [15].

Type 2 diabetes (T2DM) appears when insulin secretion is no longer sufficient to compensate for peripheral insulin resistance. This is caused by a reduced insulin secretion capacity and a reduction in the total number of beta-cells [16]. Whereas in T2DM patients GIP no longer stimulates insulin secretion GLP-1, at pharmacological concentrations, can still acutely, and glucose-dependently potentiate insulin secretion [17], [18]. Newer strategies for the treatment of T2DM therefore aim at increasing GLP-1 signaling. This approach depends on the acute stimulation of insulin secretion and it is still uncertain whether the increase in beta-cell mass and function observed in rodents also takes place in humans. Current evidence rather suggests the opposite since cessation of incretin therapy rapidly leads to re-appearance of hyperglycemia [19]. It is not clear whether the apparent absence of trophic action on human islets is due to a late initiation of the treatment when beta-cells are already severely dysfunctional or whether human beta-cells respond to gluco-incretin hormones in a different manner than rodent beta-cells. It is therefore important to better understand the molecular action of gluco-incretins on beta-cells.

In previous studies, we showed that islets from *Gipr^−/−^; Glp-1r^−/−^* (dKO) mice had reduced GIIS but normal insulin sensitivity [20], increased susceptibility to cytokine-induced apoptosis [15], and reduced glucose competence [7]. These defects were cell-autonomous and maintained when islets were maintained in *in vitro* cultures. Here, we identify *Fxyd3* as the gene that is most overexpressed in dKO islets. Fxyd3 belongs to the Fxyd family of single transmembrane domain containing proteins. These are best known as third subunits of the Na^+^/K^+^-ATPase, which can change the affinity of the pump for either Na^+^ and/or K^+^ [21]. Fxyd3, also called Mat-8 [22], has a unique topology with two transmembrane domains. It can also associate with the H^+^/K^+^-ATPase, regulate hyperpolarization-activated chloride channels in Xenopus oocytes [22], and its expression is required for the differentiation of the intestinal CaCo2 cell line [23]. It is also overexpressed and may control proliferation of different cancer types [24], [25]. In this study, we show that Fxyd3 is a negative regulator of GIIS whose expression is negatively regulated by gluco-incretin hormone-dependent promoter methylation, a control that is lost in islets from diabetic mice and humans leading to Fxyd3 overexpression.

## Research Design and Methods

### Mice

C57BL/6J and *Glp1r^−/−^; Gipr^−/−^* (dKO) mice backcrossed in C57BL/6J background were used. *Glp1r^−/−^* and *Gipr^−/−^* and dKO mice were littermates obtained by crossing *Glp1r^+/−^; Gipr^+/−^* heterozygous mice. *db/db* and *db/+* mice were from Janvier (Le Genest/Isle, France). *db/db* and *db/+* mice were 9 weeks old at the time of experiments. Mice were killed by cervical dislocation after isoflurane anesthesia. All experimental procedures received approval from the Service Vétérinaire du Canton de Vaud.

### Antibodies

Rabbit anti-mouse Fxyd3 was a gift from Pr. K. Geering (University of Lausanne). Goat anti-mouse immunoglobulin antibodies (M-20) were from Santa Cruz Biotechnology (Nunningen, Switzerland); guinea pig anti-insulin antibodies (A0564) from DAKO; rabbit anti-actin antibodies from Sigma (A2066).

### Cell Culture

MIN6 cells were from Drs Miyazaki and maintained as described [26]. For transient transfection they were seeded at 0.25.10^6^ cells per well, transfected one day later with Lipofectamine 2000 (Invitrogen, Carlsbad, CA) and used 48 h later. Stable transfection was performed with recombinant lentiviruses and G418 selection [27]. For secretion tests, 20 islets or 0.25.10^6^ MIN6 cells were placed in 12-well plates; MIN6 cells were used 4 days later. After a 2 h incubation in Krebs-Ringer bicarbonate HEPES buffer (KRBH, 120 mM NaCl, 4 mM KH2PO4, 20 mM HEPES, 1 mM MgCl2, 1 mM CaCl2, 5 mM NaHCO3, and 0.5% BSA, pH 7.4) containing 2 mM glucose the medium was replaced with KRBH containing 2 or 20 mM glucose for one hour. Insulin was determined by radioimmunoassay (Millipore, Billerica, MA, USA). Intracellular calcium concentrations recording were performed on stably transfected MIN6 cells as described [28].

### Primary islets studies

Adult islets were isolated as described [29]. Neonates islets were handpicked from collagenase-digested pancreas. Immunofluorescence microscopy analysis of islets monolayers seeded on extracellular matrix-coated plates (Novamed, Jerusalem, Israel) was performed as described [29]. Adenoviral transductions of dissociated islets (3 minutes at 37°C in a Hank’s balanced salt solution, 5 mM glucose, 1 mM EGTA) were performed with pAdGFP or pAdFxyd3 adenoviruses (AdEasy system [30]) with a multiplicity of infection of 50. Assays were performed 48 h later.

### Bisulfite sequencing

Islet DNA was extracted using the DNeasy Blood and Tissue kit from Qiagen (Hilden, Germany). 500 ng of DNA were converted using Epitect Bisulfite kit from Qiagen. Then, R1 (+84; −362) and R2 (–655; −1138) regions from the *Fxyd3* promoter were PCR amplified (for primers see Table S1) and sub-cloned into TOPO-TA vector (Invitrogen, Carlsbad, CA, USA). Ten clones per region and per mouse were then sequenced in both directions to assess CpG methylation status.

### Pyrosequencing

Six regions of the *Fxyd3* promoter were PCR amplified (Pyromark PCR kit from Qiagen) with one biotinylated primer (see Table S1) starting from 30 ng of bisulfite-treated DNA. Pyrosequencing was performed on a PSQ 96MA instrument (Qiagen) using Pyromark Gold Reagents from Qiagen. For each human sample 12 regions of the *FXYD3* promoter were PCR amplified and pyrosequenced. Primers (Table S2) were designed with the Biotage PSQ Assay Design software and data were analysed by the Pyro Q-CpG software (Qiagen).

### Chromatin Immunoprecipitation

Islets were pooled from 4 adult mice per ChIP experiment performed using minor modifications of the micro-ChIP protocol [31]. Islets were lysed with 115 µL of lysis buffer (50 mM Tris-HCl pH 8,0; 10 mM EDTA; 1% SDS; 1 mM PMSF; 20 mM butyrate; protease inhibitors cocktail from Roche) during 20 minutes at 4°C. Samples were then sonicated 3x[20 sec ON/40 sec OFF] and washed with RIPA ChIP buffer (10 mM Tris-HCl pH 7,5; 1 mM EDTA; 1% TX-100; 0,1% SDS; 0,1% Na-deoycholate; 100 mM NaCl; 1 mM PMSF; 20 mM butyrate; protease inhibitors cocktail). 1 µg of anti-H3K4me3 (Diagenode, Denville, NJ, USA) and 10 µL of agarose beads blocked with sonicated salmon sperm (Millipore, Temecula, CA, USA cat #16–157) were used per ChIP sample. After elution of DNA/Protein/antibodies complexes, reversal of the crosslinking and proteinase K/RNAse A treatment, DNA was purified using the NucleoSpin kit from Macherey-Nagel (Duren, Germany). For primers used see Table S1.

### Luciferase assay

MIN6 cells were seeded at 0.15.10^6^ cells per well in 24-well plates. The day after plating, they were co-transfected with 30 ng of Renilla luciferase vector and 750 ng of the firefly luciferase reporter plasmids using Lipofectamine 2000 (Invitrogen). Firefly and renilla luciferase activities were measured 48 h later with a Glomax Instrument (Promega, Madison, WI, USA). The mouse *Fxyd3* promoter (–731 to +19) was cloned into pGL3basic vector (Promega) or the pCpGLbasic, CpG-free vector (gift from Dr. M. Rehli, Regensburg, Germany). *In vitro* methylation was performed using SssI and S-adenosyl-methionine (SAM) (New England Biolabs, Ipswich, MA, USA).

### Human islet microarray analysis

Human islet RNA was prepared from laser capture microdissected samples and profiled by microarray analysis [32].

### Statistical analysis

All experiments were performed at least three times. Results are expressed as means +/− sem. Comparisons were performed using unpaired Student’s *t* test or one-way or two-way ANOVA for the different groups followed by post hoc pair-wise multiple-comparison procedures (Tukey test or Bonferroni, respectively).

## Results

### *Fxyd3* overexpression in islets from dKO mice

**Figure 1:**
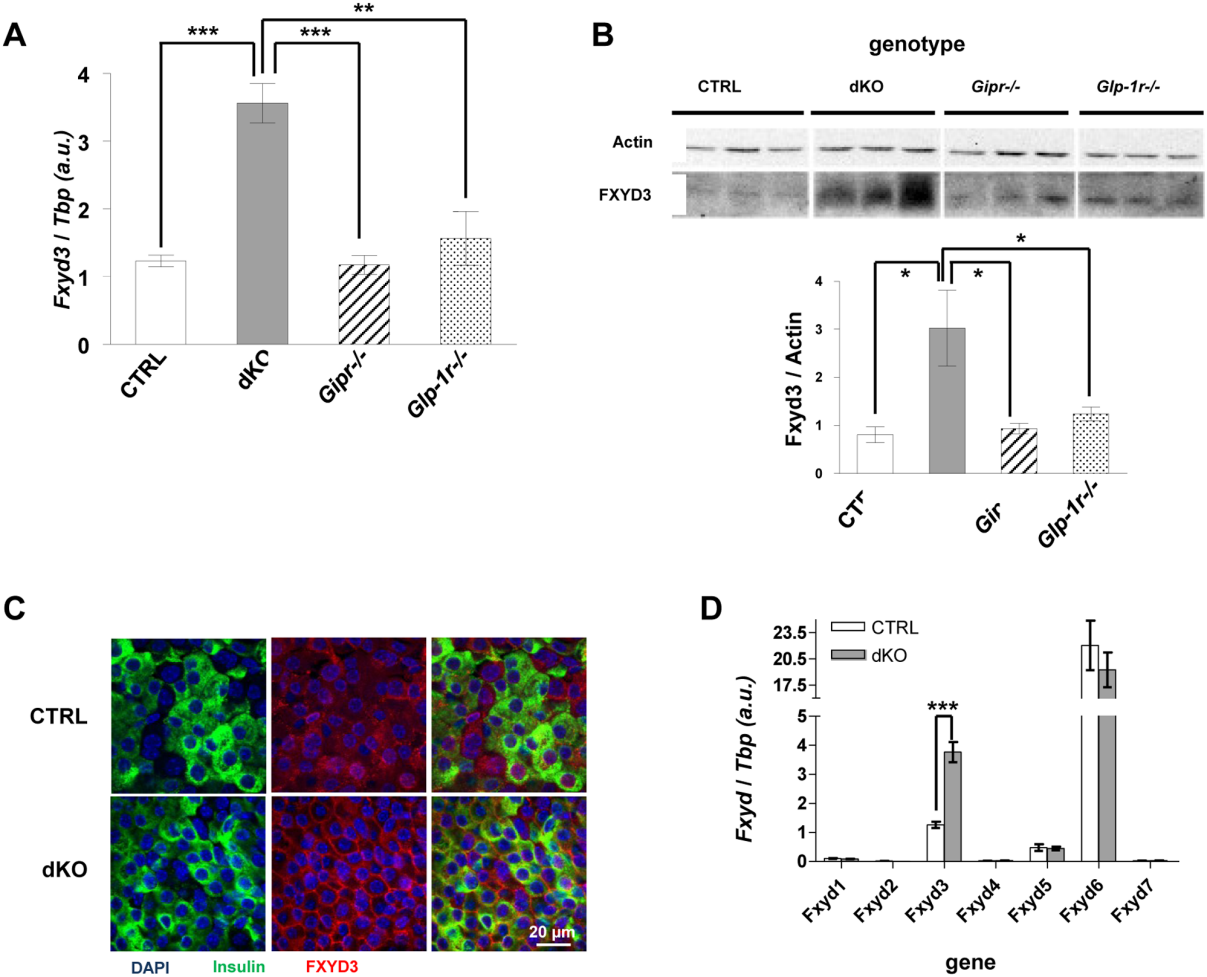
Fxyd3 is overexpressed in dKO islets. (A) *Fxyd3* mRNA level in primary islets from control (CTRL), dKO, *Glp1r^−/−^* or *Gipr^−/−^* mice. Data are mean ± sem, n = 4–5; **p<0,01, ***p<0,001. (B) Western blot analysis and quantification of Fxyd3 expression in primary islets from CTRL, dKO, *Glp1r^−/−^* and *Gipr^−/−^* mice. Data are mean ± sem, *p<0,05, n = 4–5. (C) Confocal immunofluorescence microscopy detection of insulin and Fxyd3 in CTRL and dKO islets cultured on ECM plates. (D) Quantitative RT-PCR analysis of *Fxyd* family members expression in control and dKO islets. Data are mean ± sem n = 4–5, ***p<0,001 compared to CTRL.

Comparative transcriptomic analysis of islets from control and dKO mice [15] revealed *Fxyd3* as the most up-regulated mRNA in mutant as compared to control islets (data not shown). Quantitative RT-PCR (Figure 1A) and western blot analysis (Figure 1B) confirmed a three-fold increase in Fxyd3 mRNA and protein expression in islets from adult dKO mice and showed that expression was similar in islets from control or single gluco-incretin receptor knockout mice (*Glp1r^−/−^* or *Gipr^−/−^* mice). Immunofluorescence microscopy analysis performed on dKO islet cell monolayers showed that FXYD3 expression was expressed at the cell surface (Figure 1C). Quantitative RT-PCR analysis of all the members of the *Fxyd* family revealed relatively high expression of *Fxyd6*, intermediate levels of *Fxyd3*, and very low expression of the other isoforms (Figure 1D). Importantly, only *Fxyd3* was differentially expressed in islets from control as compared to dKO mice. These data indicate that in the absence of both gluco-incretin receptors there is a selective overexpression of *Fxyd3*, which is correlated with decreased GSIS [20].

### *FXYD3* overexpression impairs glucose-stimulated insulin secretion

**Figure 2:**
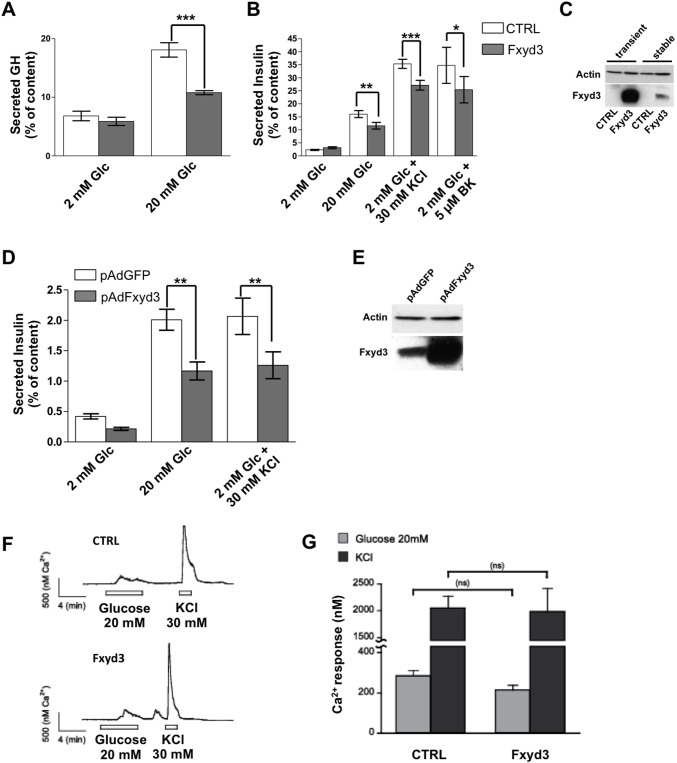
Fxyd3 overexpression impairs glucose stimulated insulin secretion in MIN6 cells and primary beta.-cells. (A) MIN6 cells were transiently transfected with a control or *Fxyd3* expression plasmid and a plasmid for expression of hGH. Secretion of hGH was then measured at the indicated glucose concentrations. Data are mean ± sem; n = 5 experiments realized in triplicates, ***p<0,001. (B) MIN6 cells stably transfected with a control or *Fxyd3* expressing construct were exposed to the indicated concentrations of glucose, KCl or the calcium channel agonist BayK8644. Insulin and hGH secretions were measured in control and *Fxyd3* over-expressing cells/islets in multiple experiments. A two-way anova with repeated measurements (pairing each experiment) with post hoc Bonferroni test was used to compare the groups. Data are mean ± sem, n = 10 experiments realized in triplicates, *p<0,05 **p<0,01 ***p<0,001. (C) Western blot analysis of Fxyd3 expression in transiently or stably transfected MIN6 cells. (D, E) Primary islets were isolated from control mice and infected either with *LacZ* or *Fxyd3* adenoviruses. Islets were challenged with the indicated glucose and KCl concentrations. Data are mean ± sem; n = 7 experiments realized in triplicates; **p<0,01). (E) Western blot analysis of Fxyd3 expression in control and Fxyd3 adenoviruses infected islets. (F and G) Intracellular calcium concentrations measured using the Fura2 ratiometric method. Stably control- or *Fxyd3*-transduced MIN6 cells were superfused with 2 or 20 mM glucose and 30 mM KCl as indicated. (J) Quantification of the calcium response. Data are mean ± sem, n = 20 per group.

To assess whether *Fxyd3* overexpression impacts on insulin secretion, we transiently co-transfected MIN6 cells with a *Fxyd3* and a human growth hormone (hGH) expression plasmids or with control vectors. The cells were then challenged with low (2 mM) or high (20 mM) glucose concentrations and hGH secretion was measured. High glucose concentrations induced a ∼3-fold increased secretion rate in control cells and this was markedly lower in *Fxyd3* overexpressing cells (Figure 2A). Separately, we established MIN6 cell lines expressing *Fxyd3* or *LacZ* by lentiviral transduction. Figure 2B shows that insulin secretion was reduced in *Fxyd3* expressing as compared to control cells when stimulated by glucose, by KCl, or by the calcium channel agonist BayK8644. The level of *Fxyd3* overexpression in transiently and stably transfected MIN6 cells is shown in Figure 2C; there was no detectable expression of FXYD3 in non-transfected MIN6 cells. When recombinant adenoviruses were used to transduce *Fxyd3* or *GPF* in mouse islets overexpression of *Fxyd3* significantly impaired glucose- as well as K^+^-induced insulin secretion (Figure 2D). Overexpression of Fxyd3 was verified by western blot analysis (Figure 2E). We next assessed whether glucose-induced raise in intracellular Ca^++^ concentrations was normal in *Fxyd3* overexpressing cells. Figure 2F, G shows that the intracellular calcium concentrations increased similarly in Ctrl and *Fxyd3* overexpressing MIN6 cells following a glucose challenge or K^+^-induced depolarization. Together, these results indicate that *Fxyd3* overexpression indeed reduces stimulated insulin secretion and that the defect in glucose signaling lays downstream of membrane depolarization and calcium entry.

### *Fxyd3* expression is not acutely regulated by the cAMP/PKA pathway

**Figure 3:**
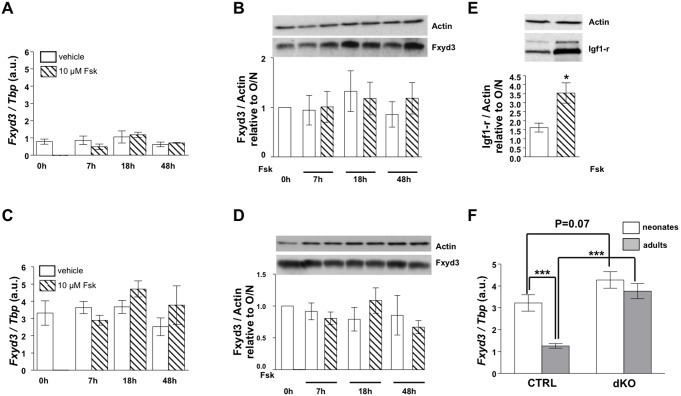
Fxyd3 expression is not regulated by exendin-4 or forskolin in adult islets. Primary islets from adult control (A, B) and dKO (C, D) mice were incubated with DMSO (vehicle) or 10 µM forskolin (fsk) for the indicated periods of time after O/N rest. Then, RNA (A, C) or proteins (B, D) were extracted for quantitative analysis of Fxyd3 expression. Data are mean ± sem, n = 3 to 4. (E) Induction of IGF1-R expression in control islets after 18 hours of forskolin treatment. (F) *Fxyd3* is down-regulated during post-natal development in control but not in dKO islets. Neonates were 3 to 4 days-old. Data are mean ± sem, n = 10 to 11 samples per group, ***p<0,001.

Since *Fxyd3* is overexpressed when both gluco-incretin receptor genes are inactivated we postulated that *Fxyd3* expression could be under negative regulation by the cAMP signaling pathway. We thus tested expression of *Fxyd3* in control or dKO islets treated with forskolin for 7, 18 or 48 hours. Neither *Fxyd3* mRNA (Figure 3A, C) nor protein (Figure 3B, D) expression was modified in these conditions whereas the expected increased expression of the IGF-1R was observed (Figure 3E). Treatment of control islets with 100 nM exendin-4 for the same periods of time did not modify Fxyd3 mRNA expression (not sown). Thus, these data indicate that activation of the cAMP/PKA pathway does not acutely regulate *Fxyd3* expression in adult islets. Instead, they suggest that absence of gluco-incretin signaling induces a permanent change in the regulation of *Fxyd3* gene expression. We thus evaluated whether the differential expression of *Fxyd3* in islets from dKO vs. control mice was already present at birth or was established during postnatal development. *Fxyd3* expression was assessed in islets of 3–4 day-old mice and compared to that of adult control and dKO mice (Figure 3F). Expression of *Fxyd3* was slightly higher in islets from neonatal dKO as compared control mice, although the difference did not reach statistical significance (p = 0.07). However, whereas *Fxyd3* expression was reduced in islets from adult as compared to neonatal control mice, no such reduction in expression was observed in the dKO islets.

Thus, *Fxyd3* expression is normally suppressed in islets during postnatal development by a gluco-incretin-dependent mechanism distinct from the cAMP/PKA/CREBP transcriptional regulatory pathway.

### Methylation of the *Fxyd3* promoter reduces gene transcription

**Figure 4:**
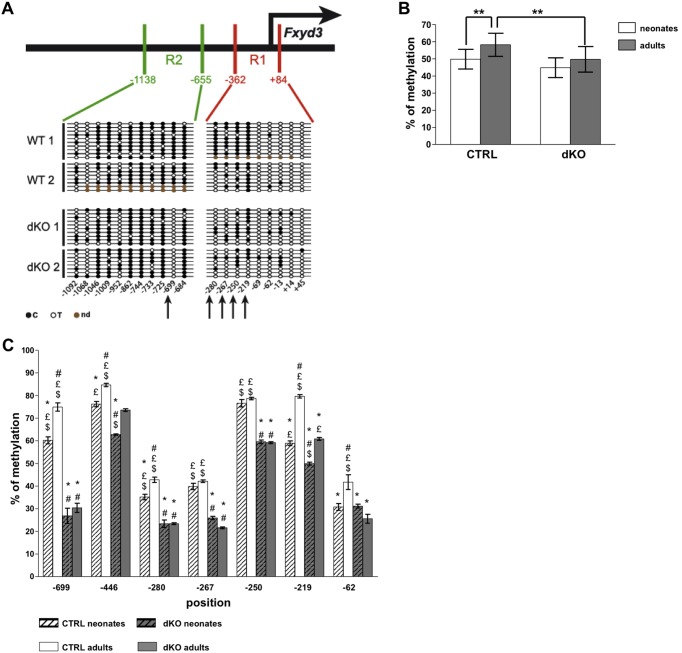
Differential methylation of the *Fxyd3* promoter in control and dKO islets. (A) Upper part: schematic representation of the *Fxyd3* promoter and the R1 and R2 regions that have been analyzed by sequencing following bisulfite conversion of cytosines. Lower part: schematic representation of the cytosines that were methylated (black dots), not methylated (circles) or not determined (brown dots). (B, C) DNA was prepared from control and dKO adult or neonates islets. Following bisulfite treatment, pyrosequencing was performed to quantitate the methylation of individual CpGs. (B) Global promoter methylation in neonate and adult islets. These data were derived from the calculated means of the percent methylated CpG at each position and for each group of mice. These means were compared for each position between groups using a two-way anova with repeated measurements with post-hoc Tukey test. Data are mean ± sem, n = 6–12 islet preparations; **p<0,01. (C) Quantitative methylation of seven CpGs in islets of control and dKO neonate and adult mice. Data are mean ± sem, n = 6–12 islet preparations; statistics are: #p<0.05 when compared to CTRL neonates (white dashed bars); *p<0.05 when compared to CTRL adults (white bars); £ p<0.05 when compared to dKO neonates (grey dashed bars) and $ p<0.05 when compared to dKO adults (grey bars).

To determine whether this regulatory events depend on *Fxyd3* promoter methylation, a mechanism of gene silencing [33], [34], we analyzed *Fxyd3* promoter methylation using bisulfite conversion of MeCpGs followed by sequencing. We analyzed two segments of the mouse *Fxyd3* promoter, the R1 region from −362 to +84 and the R2 region from −1138 to–665 (relative to the transcriptional start site +1) (Figure 4A), which were found based on sequence analysis (UCSC genome browser, http://genome.ucsc.edu/) to contain 20 out of the 21 CpGs of this proximal promoter region. Bisulfite conversion efficiency was assessed by measuring the conversion rate of cytosines located outside of CpG sequences and was found to be ∼98%. Five CpG were differentially methylated between control and dKO islets at positions −699 (81% in Ctrl vs. 0% in dKO); −280 (59% in Ctrl vs.22% in dKO); −267 (53% in Ctrl vs. 22% in dKO); −250 (71% in Ctrl vs. 53% in dKO); −219 (94% in Ctrl vs. 56% in dKO) (Figure 4A).

To get quantitative information about the differential methylation of these sites, we used pyrosequencing analysis. Following bisulfite conversion of adult and 3–4 day-old mouse islet genomic DNA, 6 regions were PCR amplified using a biotinylated primer and sequenced. Global methylation level of the *Fxyd3* promoter was the same in islets from control and dKO neonate mice (Figure 4B). This methylation level was not changed during development to the adult stage in dKO islets but was significantly increased in control islets (Figure 4B). Detailed analysis of the methylation patterns (Figure 4C) showed that seven CpGs (–699; −446; −280; −267; −250; −219; −62) were significantly more methylated in control than in dKO adult islets, and the CpGs at positions −699; −446; −280; −219; −62 were more methylated in adult as compared to neonate control islets. In dKO islets, methylation of the CpGs at position −699, −280, −267, −250, −62 was not increased when comparing islets from neonate and adult mice; only those at position −446 and −219 were increased. Thus, in the absence of gluco-incretin receptors a differential methylation of the *Fxyd3* promoter is already evident at 3–4 days of age but is fully established in adult mice.

**Figure 5:**
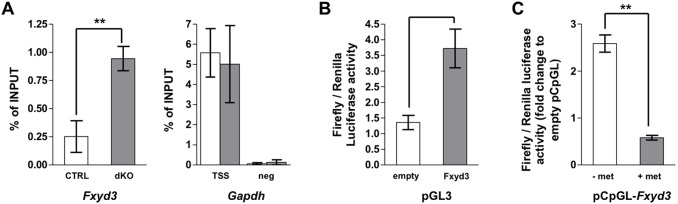
Hypermethylation of the *Fxyd3* promoter reduces transcriptional activity. (A) ChIP analysis using H3K4me3 antibody reveals enrichment in this histone mark at the transcriptional start site of the *Fxyd3* gene in dKO as compared to control islets. Results are expressed as percent of total input. Data are mean ± sem, n = 3 experiments, *p<0,05. (B) Luciferase activity measured in MIN6 cells transfected with a basic pGL3 or a *Fxyd3*promoter-PGL3 reporter construct. Data are mean ± sem, n = 7 experiments realized in triplicates, ***p<0,001. (C) The same sequence of the mouse *Fxyd3* promoter was sub-cloned into the pCpGL vector (free of CpG). Following in vitro methylation (grey bar) or mock treatment (white bar), basic or *Fxyd3*promoter-pCpGL plasmids were transfected into MIN6 cells and luciferase activity was measured 48 h later. Methylation significantly reduces luciferase activity. Data are mean ± sem, n = 3 experiments realized in triplicates, **p<0,01. (B, C) Plasmids were co-transfected each time with Renilla reporter vector for normalization.

To determine whether the level of methylation of the *Fxyd3* promoter correlates with transcriptional activity, we determined the degree of association of H3K4me3, a mark of actively transcribed genes [35], with the transcription start site (TSS) of the *Fxyd3* gene using chromatin immunoprecipitation (ChiP) assays. Figure 5A shows a higher presence of H3K4me3 at the TSS of the *Fxyd3* gene in islets of dKO as compared to control mice. Association of H3K4me3 with the *Gapdh* TSS was the same in both types of islets. Thus lower methylation of the *Fxyd3* promoter in dKO islets correlated with increased transcriptional activity.

To get an independent evidence for the role of promoter methylation in gene expression, we constructed a luciferase transcriptional reporter plasmid. This comprised the −731 to +19 segment of the *Fxyd3* promoter subcloned in front of the minimal promoter of the PGL3 firefly luciferase vector. This part of the *Fxyd3* promoter contains all the differentially methylated CpGs. When transfected in MIN6 cells, this reporter construct induced a three-fold stimulation of luciferase activity (Figure 5B). To determine whether methylation of this promoter sequence would reduce transcriptional activity, we first sub-cloned the *Fxyd3* promoter sequence into the CpG-free vector pCpGLbasic. This plasmid was then methylated in vitro and transfected into MIN6 cells. Methylation markedly decreased luciferase activity (Figure 5C).

### *Fxyd3* promoter methylation upon gluco-incretin treatment and in diabetes in mouse and human islets

**Figure 6:**
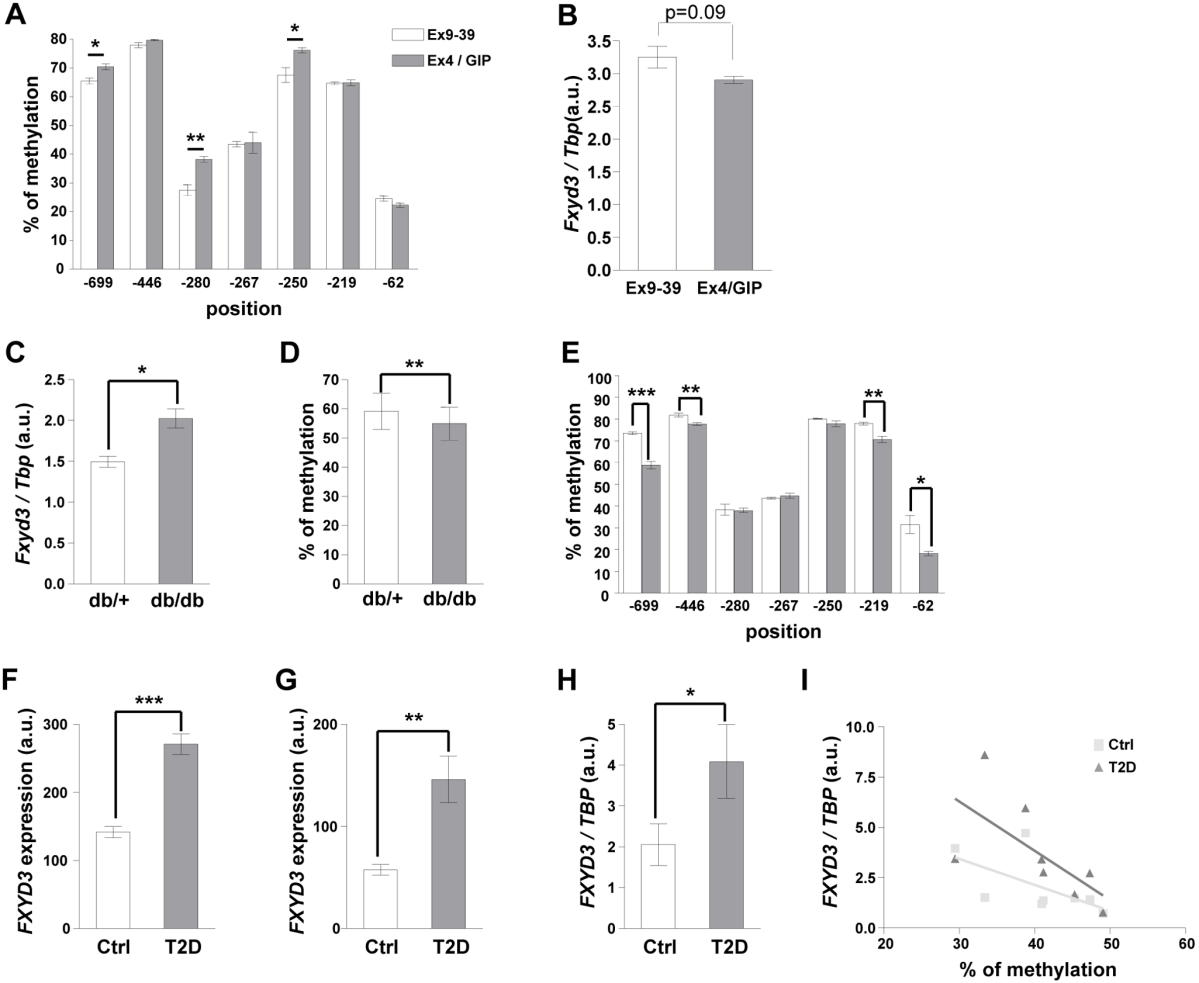
*Fxyd3* promoter methylation in neonates and in diabetic mouse and human islets. (A) Ctrl neonates received daily injections for 7 days of Ex9–39 (2,5 nmol/kg) or Ex4 (2,5 nmol/kg) and GIP (80,4 nmol/kg). Methylation of CpG sites was determined by pyrosequencing. Data are mean ± sem, n = 3 experiments, *p<0,05, **p<0,01 as compared to control. (B) *Fxyd3* mRNA expression in islets prepared as in (A). Data are mean ± sem, n = 4 experiments. (C) *Fxyd3* mRNA expression in islets from *db/+* and *db/db* mice. These data were derived from the calculated means of the percent methylated CpG at each position and for each group of mice. These means were compared for each position between groups using a paired t-test. Data are mean ± sem, n = 4, *p<0,05. (D) Pyrosequencing analysis of global *Fxyd3* promoter methylation in islets from *db/+* and *db/db* mice. Data are mean ± sem, n = 4, **p<0,01. (E) Pyrosequencing analysis of the methylation of individual CpGs. Data are mean ± sem, n = 4, *p<0,05, **p<0,01, ***p<0,001. (E, F) *FXYD3* expression in human islets assessed by Affymetrix chip analysis (F), probeset g13528881_3p_a_at; (G), probeset g11612675_3p_a_at. Data are mean ± sem, n = 10 per group, **p<0,01 and ***p<0,001 compared to controls. (H) *FXYD3* mRNA expression measured by real-time PCR using a separate set of control (n = 8 donors) and T2D (n = 8 donors) humans islets. Data are mean ± sem, *p<0,05. (I) Inverse correlation of CpG -43 methylation and Fxyd3 expression in islets from Ctrl and T2DM patients.

To get a direct assessment of the role of gluco-incretin in the regulation of *Fxyd3* promoter methylation in the neonates, we injected d0 control mice once a day for seven consecutive days with either exendin-9-39 to antagonize GLP-1 action, or with a combination of exendin-4 and GIP. As shown in Figure 6A, gluco-incretin treatment increased methylation of the CpGs at position −699, −280, and −250. Thus, *in vivo* administration of gluco-incretins in neonate mice further increased *Fxyd3* promoter methylation at sites that are differentially methylated between control and KO islets, indicating a direct role of these hormones in the epigenetic regulation of Fxyd3. This increased methylation lead to a slight but not significant decreased of *Fxyd3* mRNA expression (Figure 6B, p = 0,09).

Next we determined *Fxyd3* expression and promoter methylation in islets from *db/+* and *db/db* mice. *Fxyd3* mRNA was overexpressed and pyrosequencing analysis showed significantly lower global methylation of the *Fxyd3* promoter in islets from *db/db* as compared to *db/+* mice (Figure 6C, D). When focusing on the seven CpGs analyzed above (Figure 4D), we found that 4 of them (at positions −699; −446; −219 and −62) showed decreased methylation in diabetic conditions (Figure 6E). Thus, an increase in *Fxyd3* expression, caused by promoter undermethylation, may explain part of the secretion defect.

Finally, to determine whether *Fxyd3* was also expressed in human islets and whether its expression was regulated in type 2 diabetes, we assessed *Fxyd3* expression by microarray and qRT-PCR analysis. Figure 6F, G shows that *Fxyd3* expression, determined by microarray analysis using two different probesets, was increased in islets from type 2 diabetic patients as compared to control individuals. This was confirmed by quantitative PCR analysis using a separate set of diabetic and control islets (Figure 6H). For each of the 32 CpGs (Table S2) found in the 1.2 kb proximal promoter of human *Fxyd3* we analyzed the correlation between their methylation and the level of *Fxyd3* mRNA expression. This analysis yielded significant correlation for 7 of these CpGs, all located in the proximal promoter region (–535 to −43) (Table 1). Importantly, 2 of these methylated CpGs, at positions −177 and −43, were highly correlated with Fxyd3 expression only in diabetic islets (Figure 6I and Table 1).

**Table 1:** Correlation between *FXYD3* expression and percent of methylation of individual CpGs.

	All subjects		Controls		T2D patients	
CpGposition	r coeff	p value	r coeff	p value	r coeff	p value
–43	–0,7219	0,0016	–0,7143	0,0576	–0,9048	0,0046
–177	–0,7353	0,0012	–0,6667	0,0831	–0,9286	0,0022
–197	–0,5588	0,0244				
–230	–0,6265	0,0094				
–391	–0,5412	0,0304				
–452	–0,5029	0,0471				
–535	–0,5971	0,0146				

DNA from control (n = 8) and T2D (n = 8) human islets were extracted from the same samples as used for the real-time PCR analysis. Then, the 32 CpGs of the *FXYD3* promoter from −1 to −1200 were analyzed by pyrosequencing. Correlation analysis between methylation level and *FXYD3* expression measured by quantitative RT-PCR was performed for each CpG using the non-parametric Pearson test. 7 CpG sites in the proximal *FXYD3* promoter showed significant correlation between methylation level and *FXYD3* expression. Within these 7 CpGs, 2 sites showed strongly significant correlation between methylation and *FXYD3* mRNA expression only in the T2D group.

## Discussion

Here, we show that gluco-incretin hormones regulate the expression of *Fxyd3*, a newly identified regulator of beta-cell glucose competence, by controlling the methylation its promoter. This epigenetic imprinting is established perinatally and partially lost in glucose incompetent islets from diabetic mice and humans, which display increased expression of *Fxyd3*. Thus, gluco-incretin action early in life contributes to the establishment of the normal insulin secretion capacity of adult islets; loss of this imprinting may contribute to the pathogenesis of type 2 diabetes.

Fxyd3 is a member of the FXYD family of proteins known to regulate ion transporting membrane proteins, which can modulate cellular differentiation, and whose expression is strongly up-regulated in some tumors, making it a good cancer biomarker [21], [23] [36]–[39]. Why overexpression of Fxyd3 in beta-cells reduces glucose-stimulated insulin secretion is not yet know. This may be based on the interaction of Fxyd3 with membrane proteins controlling beta-cell electrical activity or insulin granule exocytosis. Additional work will clearly be required to answer this question.

We found Fxyd3 to be overexpressed in islets from dKO mice and to negatively regulate glucose competence of insulin secreting cells. Due to the limited information available on Fxyd3 function, we further focused our attention on the unexpected mechanism by which gluco-incretins regulate *Fxyd3* expression. Indeed, forskolin treatment, which induces marked accumulation of cAMP in primary beta-cells, did not impact *Fxyd3* expression in dKO nor in control islets. Second, overexpression of *Fxyd3* in dKO islets was cell-autonomous and maintained in *in vitro* cultured islets indicating a permanent change in gene expression. Thus, the classical cAMP/PKA signaling pathway that acutely controls gene expression through phosphorylation of CREBP was unlikely to be involved in regulating Fxyd3 expression in adult islets. In neonatal islets, *Fxyd3* expression was slightly higher in dKO than in control islets and this initial level of expression was maintained in islets from adult dKO mice but markedly reduced in those of control mice. We therefore suspected that absence of gluco-incretin signaling involved a change in epigenetic control of *Fxyd3* expression. Analysis of *Fxyd3* promoter methylation revealed differential methylation of seven CpGs in islets from adult control as compared to dKO mice. Chromatin immunoprecipitation analysis showed enrichment of H3K4me3 at the transcriptional start site of *Fxyd3* in dKO islets confirming higher transcriptional activity. The inverse relationship between promoter methylation and transcription rate was further supported by the *Fxyd3* promoter reporter assays.

In neonatal islets, the difference in *Fxyd3* promoter methylation was already present at most sites. Importantly, however, the difference in CpG methylation in control vs. dKO islets markedly increased during the neonatal to adult transition at positions −699, −280, −219, −62, suggesting that methylation of these sites strongly influenced *Fxyd3* transcription rate. Thus, this gluco-incretin-dependent methylation of the *Fxyd3* promoter takes place in the perinatal period and is fully established in the adult animals. With respect to the timing of this methylation events, it is interesting to note that intestinal GLP-1 and GIP producing cells appear first at embryonic day 15 (E15), rapidly increase in number at E17, with both peptides being often co-expressed in the same cells [40]. In addition, GLP-1 receptor has an intrinsic signaling activity even in the absence of ligand [41]. Thus, gluco-incretins and their receptors can influence beta-cells during embryonic development and in the perinatal period. In addition, since these methylation events occur perinatally in response to gluco-incretin action, changes in hormonal and nutritional status during pregnancy or early in life may have long-term impact on beta-cell function and the susceptibility to develop diabetes in the adult age.

Epigenetic regulation of gene expression by nutrition and metabolic status is known to modulate the activity of multiple cellular pathways [42]. Nutrition during pregnancy can impact gene expression in offsprings through changes in DNA methylation [43] and similar effects of nutrition during the postnatal period on the susceptibility to develop metabolic disease in the adult age has also been linked to epigenetic modifications [44]. In humans, it has been shown that islets from type 2 diabetic patients display numerous changes in gene methylation patterns [45], and that, in muscle, diabetes is associated with hypermethylation of the *Pgc1Α* promoter and lower gene expression leading to reduced mitochondrial content [46]. Thus, whereas it is well established that DNA methylation regulates gene expression, our study uncovers a so far unrecognized role of gluco-incretins in epigenetic regulation of gene expression that takes place early in life. This could explain preceding observations that administration of GLP-1 to diabetes-prone rats during the first week of life protected them against the development of diabetes in their adult life [47], [48]. Although the mechanism of this protection was not established, it may involve epigenetic control of beta-cell function as reported here.

Interestingly, we found that *Fxyd3* expression was also increased in glucose-unresponsive beta-cells from diabetic mice and humans and that this was correlated with reduced methylation of the *Fxyd3* promoter. In *db/db* mice three of the four sites that were differentially methylated during maturation of adult islets (positions −699, −219 and −62), and which we propose may have a particularly important role in controlling *Fxyd3* expression, displayed significantly reduced methylation. This suggests that the methylation of these sites may be dynamically controlled by gluco-incretin action. Indeed, type 2 diabetes is characterized by beta-cell gluco-incretin resistance [49]–[51], which may explain the reduced methylation and increased expression of *Fxyd3*. Alternatively, it has been reported that diabetic hyperglycemia caused beta-cell dedifferentiation as revealed by over expression of transcription factors and enzymes normally present in precursor cells [52], [53]. Our data suggest that this may be accompanied by changes in DNA methylation.

It is not yet clear how gluco-incretins control DNA methylation. In mammals three DNA methyl transferases (Dnmts) catalyze the addition of methyl groups on CpGs [33]. Dnmt1 is responsible for propagation of methylation patterns through cell division cycles. In beta-cells, this enzyme is also required to silence the expression of the transcription factor Arx to prevent their differentiation into alpha cells [54]. Dnmt3a and 3b catalyze *de novo* methylation. Dnmts expression levels did not differ significantly in islets from dKO or Ctrl mice (not shown). Thus, a modulation of their activity by posttranslational modifications, as reported for Dnmt1 [55], [56], rather than changes in expression levels may control the methylation patterns.

In summary, we have identified Fxyd3 as a novel regulator of beta-cell glucose competence. We showed that its expression is controlled at the transcriptional level by gluco-incretin hormone-dependent methylation of its promoter. This epigenetic regulation is initiated in the perinatal period. As secretion of gluco-incretin hormones is controlled by nutrients, the mechanism we describe may link changes in early nutrition to long-term control of beta-cell function. Because this epigenetic regulation is reversed in glucose-unresponsive islets from diabetic mice and humans, which express higher levels of Fxyd3, loss of imprinting of this locus may contribute to beta-cell dysfunction characteristic of type 2 diabetes.

## Supporting Information

Table S1 **Primers list for mouse** ***Fxyd3*** **promoter analysis.**
(DOCX)

Table S2 **Primers list for human** ***Fxyd*** **promoter analysis.**
(DOCX)
