# Correction: Gluco-Incretins Regulate Beta-Cell Glucose Competence by Epigenetic Silencing of *Fxyd3* Expression

**DOI:** 10.1371/journal.pone.0116451

**Published:** 2014-12-26

**Authors:** The PLOS ONE Staff

The fourth author’s name is spelled incorrectly. The correct name is: Vini Nagaraj. The correct citation is: Vallois D, Niederhäuser G, Ibberson M, Nagaraj V, Marselli L, et al. (2014) Gluco-Incretins Regulate Beta-Cell Glucose Competence by Epigenetic Silencing of *Fxyd3* Expression. PLoS ONE 9(7): e103277. doi:10.1371/journal.pone.0103277
