# Supplementary material for: Gluco-Incretins Regulate Beta-Cell Glucose Competence by Epigenetic Silencing of Fxyd3 Expression
Source: PLoS One. 2014 Jul 24;9(7):e103277. doi: 10.1371/journal.pone.0103277 (PMC4110016; doi:10.1371/journal.pone.0103277)
Supplement: Table S1 — Primers list for mouse Fxyd3 promoter analysis. (DOCX) [file pone.0103277.s001.docx]

**Table S1: primers list for mouse *Fxyd3* promoter analysis**

**Table S1: Primers list, mouse.** 1^st^ panel: primers used for bisulfite sequencing of the R1 and R2 regions of the mouse *Fxyd3* promoter. 2^nd^ panel: primers used for targeted pyrosequencing of the mouse *Fxyd3* promoter. 3rd panel: primers used for real-time PCR measurement of *Fxyd* genes expression. 4^th^ panel: primers used for cloning the *Fxyd3* promoter region [-731; +19].
