# Supplementary material for: Gluco-Incretins Regulate Beta-Cell Glucose Competence by Epigenetic Silencing of Fxyd3 Expression
Source: PLoS One. 2014 Jul 24;9(7):e103277. doi: 10.1371/journal.pone.0103277 (PMC4110016; doi:10.1371/journal.pone.0103277)
Supplement: Table S2 — Primers list for human Fxyd promoter analysis. (DOCX) [file pone.0103277.s002.docx]

**Table S2: primers list for human *Fxyd* promoter analysis**

**Table S2: Primers list, human.** 1^st^ panel: primers used for targeted pyrosequencing of the human *FXYD3* promoter. 2^nd^ panel: primers used for real-time PCR measurement of *FXYD3* expression in human islets.
